# Polyspermy Block in the Central Cell During Double Fertilization of *Arabidopsis thaliana*

**DOI:** 10.3389/fpls.2020.588700

**Published:** 2021-01-12

**Authors:** Shiori Nagahara, Hidenori Takeuchi, Tetsuya Higashiyama

**Affiliations:** ^1^Division of Biological Science, Graduate School of Science, Nagoya University, Nagoya, Japan; ^2^Institute of Transformative Bio-Molecules, Nagoya University, Nagoya, Japan; ^3^Institute for Advanced Research, Nagoya University, Nagoya, Japan; ^4^Department of Biological Sciences, Graduate School of Science, The University of Tokyo, Tokyo, Japan

**Keywords:** *Arabidopsis thaliana*, double fertilization, laser irradiation, live cell imaging, polyspermy block, *tetraspore*

## Abstract

During double fertilization in angiosperms, two male gametes (sperm cells), are released from a pollen tube into the receptive region between two female gametes; the egg cell and the central cell of the ovule. The sperm cells fertilize the egg cell and the central cell in a one-to-one manner to yield a zygote and an endosperm, respectively. The one-to-one distribution of the sperm cells to the two female gametes is strictly regulated, possibly via communication among the four gametes. Polyspermy block is the mechanism by which fertilized female gametes prevent fertilization by a secondary sperm cell, and has been suggested to operate in the egg cell rather than the central cell. However, whether the central cell also has the ability to avoid polyspermy during double fertilization remains unclear. Here, we assessed the one-to-one fertilization mechanism of the central cell by laser irradiation of the female gametes and live cell imaging of the fertilization process in *Arabidopsis thaliana*. We successfully disrupted an egg cell within the ovules by irradiation using a femtosecond pulse laser. In the egg-disrupted ovules, the central cell predominantly showed single fertilization by one sperm cell, suggesting that neither the egg cell nor its fusion with one sperm cell is necessary for one-to-one fertilization (i.e., monospermy) of the central cell. In addition, using *tetraspore* mutants possessing multiple sperm cell pairs in one pollen, we demonstrated that normal double fertilization was observed even when excess sperm cells were released into the receptive region between the female gametes. In ovules accepting four sperm cells, the egg cell never fused with more than one sperm cell, whereas half of the central cells fused with more than one sperm cell (i.e., polyspermy) even 1 h later. Our results suggest that the central cell can block polyspermy during double fertilization, although the central cell is more permissive to polyspermy than the egg cell. The potential contribution of polyspermy block by the central cell is discussed in terms of how it is involved in the one-to-one distribution of the sperm cells to two distinct female gametes.

## Introduction

The unique reproductive system of angiosperms is known as double fertilization. One pollen grain produces two male gametes (sperm cells), which are delivered to an ovule by elongation of the pollen tube. After the pollen tube has arrived at an embryo sac, two sperm cells are discharged from the pollen tube, and these fertilize two distinct female gametes (an egg cell and a central cell) which develop into a zygote and its nutritious tissue, an endosperm. In contrast to the fertilization process of animals in which an egg is exposed to numerous sperms, the pollen tube-ovule ratio is ensured to be one-to-one by a mechanism known as polytubey block (Maruyama et al., 2013; Völz et al., 2013). Nevertheless, polyspermy block, which is the strategy employed by the female gamete(s) to avoid multiple fertilization with supernumerary sperm cells, could contribute to the strict one-to-one distribution of two sperm cells for successful double fertilization (Hamamura et al., 2012). Sperm cells are released into a narrow receptive region between the egg cell and the central cell upon double fertilization, and communication between the four gametes (namely the egg cell, the central cell, and two sperm cells) is proposed as a mechanism to determine their fertilization partners (Hamamura et al., 2011; Huang et al., 2015). Hetero-fertilization of the two female gametes suggests the potential block of additional sperm cells by fertilized female gametes because it should be caused by sperm cells that are delivered by multiple pollen tubes (polytubey) (Mogensen, 1978; Spielman and Scott, 2008; Maruyama et al., 2013; Grossniklaus, 2017). The timing of additional sperm delivery by polytubey is assumed to be simultaneous or several hours later, but remains to be clarified (Kasahara et al., 2012; Grossniklaus, 2017).

Cytological and genetic studies have demonstrated polyspermy block in the egg cell of angiosperms (Spielman and Scott, 2008; Tekleyohans et al., 2017). For example, *in vitro* fertilization studies using isolated egg cells and sperm cells of maize (*Zea mays*) revealed that the fertilized egg cell is unable to fuse with additional sperm cells, possibly by cell wall formation following a calcium ion influx (Faure et al., 1994; Kranz et al., 1995; Antoine et al., 2000). Although triploid plants can be generated *in vitro* and *in planta* in several plant species due to polyspermy of the egg cell, they rarely occur under natural conditions (Toda et al., 2016; Grossniklaus, 2017; Nakel et al., 2017; Mao et al., 2020). Meanwhile, the polyspermy block mechanism in the central cell has not been studied directly to date. Nevertheless, polyspermy block in the central cell is important because a paternal excess of genome dosage in the endosperm leads to abnormal seed development and seed abortion (Scott et al., 1998; Köhler et al., 2010). Using the *tetraspore* (*tes*) mutant of *Arabidopsis thaliana*, with pollen that possesses more than one pair of sperm cells (Spielman et al., 1997), Scott et al. (2008) examined the karyotype of seedlings and endosperm fertilized by the *tes* mutant and showed that the central cell, and not the egg cell, showed evidence of accepting multiple sperm cells. However, due to the lack of a live imaging study, it remains unclear whether the central cell also has the ability to block polyspermy, albeit not perfectly, which would allow proper and efficient double fertilization.

Several factors involved in proper double fertilization have been reported in *A. thaliana*. Sperm membrane proteins GAMETE EXPRESSED 2 (GEX2) and GENERATIVE CELL SPECIFIC 1 (GCS1)/HAPLESS 2 (HAP2) are required for gamete attachment and fusion, respectively (Mori et al., 2006, 2014; von Besser et al., 2006). Similarly, EC1 peptides, which are secreted cysteine-rich peptides from the egg cell, are essential for gamete fusion by redistributing GCS1/HAP2 on the sperm cell surface (Sprunck et al., 2012). Recently, the sperm cell membrane proteins DUF679 MEMBRANE PROTEIN (DMP) 8 and DMP9 were identified (Takahashi et al., 2018; Cyprys et al., 2019). The lack of these proteins disturbs fertilization preferentially with the egg cell. A *glauce* mutant female exhibited a biased fertilization defect specifically within the central cell (Leshem et al., 2012). However, no female mutants specifically defective in egg cell fertilization are available (Sprunck, 2020). Therefore, genetic investigation of the communication between two sperm cells and the central cell, independent of egg cell fertilization *in vivo*, is difficult.

In this study, we used a femtosecond pulse laser combined with live cell analysis. First, we specifically damaged the egg cell within the *A. thaliana* ovule and investigated the fertilization pattern in the ovule. We showed that the central cell predominantly fused with only one of the two sperm cells despite the loss of the egg cell, which was the second fertilization target. Second, we demonstrated that excessive sperm cells from *tes* pollen did not necessarily fertilize the central cell and that normal double fertilization occurred frequently. Taken together, these results suggest the existence of limited polyspermy block in the central cell.

## Materials and Methods

### Plant Materials and Growth Conditions

*Arabidopsis thaliana* Columbia (Col-0) accession was used as a wild-type control. Transgenic plants possessing *HTR10p::HTR10-mRFP* or *-Clover* (Ingouff et al., 2007; Kawashima et al., 2014), *RPS5Ap::H2B-GFP* or *-tdTomato* (Adachi et al., 2011), and *RPS5Ap::tdTomato-LTI6b* (Mizuta et al., 2015) genes were used to visualize the sperm cell nuclei, female gametophytic cell nuclei, and female gametophytic cell membrane, respectively. A *tetraspore* (*tes*) mutant, *tes-4* (CS9353; Spielman et al., 1997; Yang et al., 2003) was obtained from the Arabidopsis Biological Resource Center and crossed with the pollen from the *HTR10p::HTR10-mRFP* line.

*A. thaliana* seeds were sterilized in a solution containing 2% Plant Preservative Mixture^TM^ (Cosmo Bio, Tokyo, Japan), 50 μg/mL magnesium sulfide, and 0.1% Tween 20 for several days at 4°C. The seeds were sown on Murashige and Skoog (MS) medium [1 × MS salt (Duchefa Biochemie, Haarlem, Netherlands), 2% sucrose, 1 × Gamborg's vitamin solution (Sigma, St. Louis, MO, USA)] solidified with 0.3% Gelrite (Wako, Osaka, Japan) and adjusted to pH 5.7 with KOH. Plants were germinated and grown in a growth chamber at 22°C with continuous light. Two-week-old seedlings were transferred to soil and grown at 22°C in a plant growth room.

### Semi-*in vivo* Fertilization Assay and Laser Irradiation of Each Female Gametophytic Cell Nucleus

For the semi-*in vivo* fertilization assay, pollen tubes were grown as described previously (Nagahara et al., 2015). The pollen germination medium consisted of 0.01% (w/v) boric acid, 5 mM CaCl_2_, 5 mM KCl, 1 mM MgSO_4_, and 10% (w/v) sucrose, adjusted to pH 7.5 with KOH and solidified with 1.5% (w/v) low gelling temperature agarose (NuSieve GTG Agarose; Lonza, Basel, Switzerland) (Boavida and McCormick, 2007) or 0.001% (w/v) boric acid, 1.27 mM Ca(NO_3_)_2_, 0.4 mM MgSO_4_, 14% (w/v) sucrose, adjusted to pH 7.0 with KOH and solidified with 1.5% (w/v) ultra-low gelling temperature agarose (Type IX-A; Sigma) (Palanivelu and Preuss, 2006).

**Figure 1:**
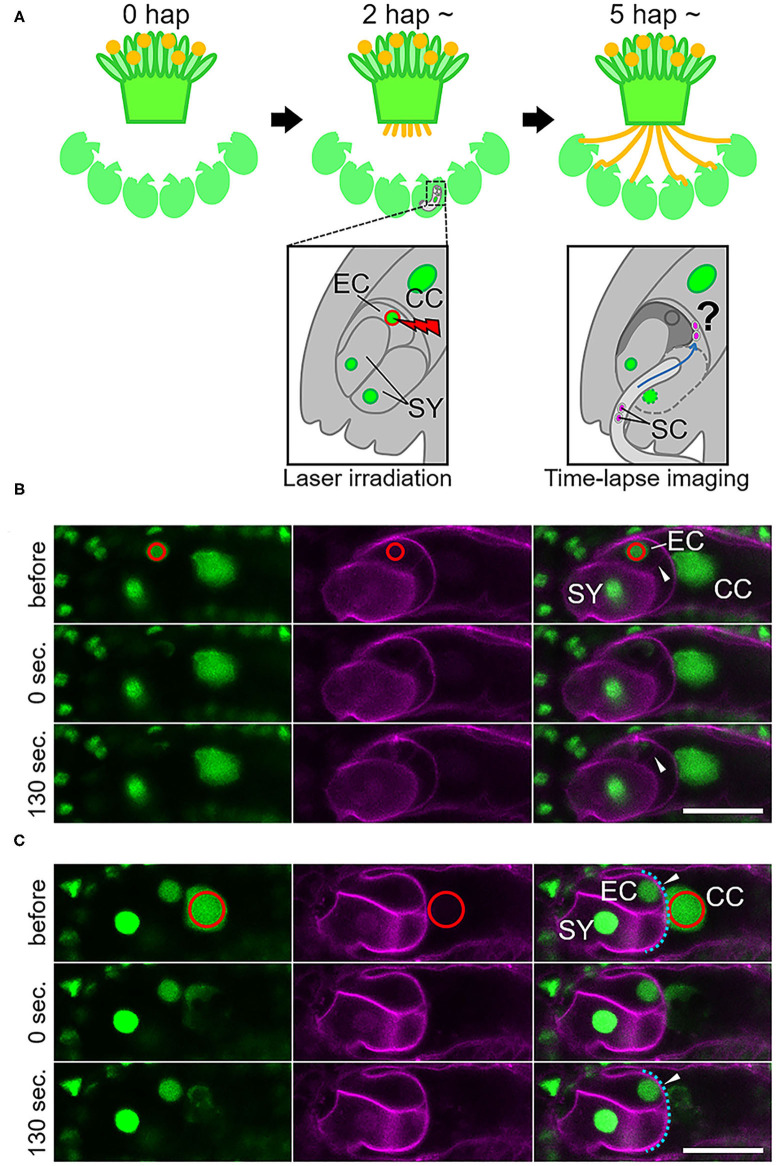
Specific damage to the egg cell or the central cell in the ovule by laser irradiation. **(A)** Schematic representation of the semi-*in vivo* fertilization assay and laser irradiation of the egg cell. A pollinated stigma and ovules were arranged on pollen germination medium. At approximately 2 h after pollination (hap), when pollen tubes emerged from the cut end of the stigma, an egg cell nucleus in each ovule was irradiated by a femtosecond pulse laser. At around 5 hap, pollen tubes reached the irradiated ovules, and sperm cell movement was examined by time-lapse imaging. EC, egg cell; CC, central cell; SY, synergid cell; SC, sperm cell. **(B,C)** A part of the embryo sac fluorescently labeled with GFP (nuclei, left) and tdTomato (plasm membrane, middle) before, just after (0 s), and 130 s after irradiation. Red circles (drawn in the top panels) indicate the laser-irradiated region, consistent with the egg cell nucleus in **(B)** and the central cell nucleus in **(C)**. Arrowheads in **(B)** indicate intact (before) and deformed (130 s) egg cell membranes. Arrowheads in **(C)** indicate intact central cell membranes (blue dashed line). Scale bars, 20 μm. EC, egg cell; CC, central cell; SY, synergid cell. See also Supplementary Figure 1 and Supplementary Videos 1, 2.

Laser irradiation was performed at 2–3 h after pollination (Figure 1). A multi-photon laser (Chameleon Vision II; Coherent, Santa Clara, CA, USA) equipped with a confocal microscope (LSM780-DUO-NLO; Zeiss, Oberkochen, Germany) was used for the laser irradiation of individual female gametophytic cell nuclei. The targeted nucleus was irradiated with an 880-nm laser 1–17 times at 70–100% of power output. After irradiation, deformation of the targeted cell was observed by a time-series acquisition at 10-s intervals for 130 s. Images were acquired and analyzed using ZEN 2010 software (Zeiss). After 5–6 h following pollination (and 2–3 h after irradiation) at 22°C in the dark, pollen tube discharge and behavior of the sperm cells were observed by spinning disc confocal microscopy as described in the next section. Just before starting time-lapse imaging, condition of irradiated ovules was checked. Ovules with uncollapsed embryo sacs outlined with autofluorescence, which is clearly observed at the boundary between the central cell and nucellus cells, were chosen for the live cell imaging.

Four categories of fertilization, i.e., double fertilization, single fertilization, polyspermy, failure of double fertilization, were judged by movies of each sample as previously described (Hamamura et al., 2011, 2014). In brief, sperm nuclei start to move toward respective target gamete nuclei when membrane fusion occurs. Mutant sperm cells of *tes* have been suggested to show increased ploidy levels (Spielman et al., 1997). We cannot exclude the possibility that sperm cells with increased ploidy behave differently, however, no difference of behavior was observed in our condition as far as two sperm cells (a pair of sperm cells) were delivered by *tes* pollen tubes.

### Microscopy Settings

Microscopy settings and image processing for the live cell imaging of semi-*in vivo* fertilization was performed as described previously (Maruyama et al., 2013; Hamamura et al., 2014; Gooh et al., 2015; Nagahara et al., 2015). To observe the laser-irradiated ovules, confocal images were acquired using an inverted microscope (IX-81; Olympus, Tokyo, Japan) equipped with water-immersion or silicone oil immersion objective lenses (UAPO 40XW3/340 or UPLSAPO60XS; Olympus) and a disk-scan confocal system (CSU-X1; Yokogawa Electric, Tokyo, Japan). From around 5 to 6 h after pollination, time-lapse and z-stack images were acquired every 5 min and on seven planes (at 3-μm intervals). The green fluorescent protein (GFP**)** and monomeric red fluorescent protein (mRFP) were excited by 488- or 561-nm diode lasers (Sapphire; Coherent), respectively, and captured with an electron multiplying CCD camera (Evolve 512; Photometrics, Tucson, AZ, USA). Maximum-intensity projection images were processed by MetaMorph version 7.7.7.0 (Universal Imaging Corp., Downingtown, PA, USA) and displayed as pseudo color images (green, GFP; magenta, mRFP).

For the live cell imaging using the *tes* mutants, confocal images were acquired using an inverted microscope (IX-83; Olympus) equipped with a silicone oil immersion objective lens (UPLSAPO60XS; Olympus), a disk-scan confocal system (CSU-W1; Yokogawa Electric), and a Piezo z-drive (P-721; Physik Instrumente, Karlsruhe, Germany). From approximately 6 h after pollination, time-lapse and z-stack images were acquired every 5 min and in seven to nine planes (at 3-μm intervals). GFP and Clover or mRFP and tdTomato were excited by 488- or 561-nm diode lasers (Sapphire; Coherent), respectively, and captured with an electron multiplying CCD camera (iXon3 888; Andor Technology, Belfast, UK). Maximum-intensity projection images were processed by MetaMorph version 7.8.4.0 (Universal Imaging Corp.) and displayed as pseudo color images (green, GFP and Clover; magenta, mRFP and tdTomato). The images and videos were edited by MacBioPhotonics ImageJ software. Two *tes* mutant alleles (*tes-4* and *tes*^*cri*^) described in the next section were used for the live cell imaging.

### Production of the *Tetraspore* Mutant via the CRISPR/Cas9 System

In addition to *tes-4* (Ws-2 accession), knockout mutants for a *TETRASPORE* (*TES*) gene (*At3g43210*) were generated by the clustered regularly interspaced short palindromic repeats (CRISPR) associated protein 9 (Cas9)-mediated genome editing system in a Col-0 background. A CRISPR/Cas9 vector targeting the *TES* gene (pSN-89) was generated based on the pKAMA-ITACHI vector (Tsutsui and Higashiyama, 2017). The construct was introduced into transgenic plants possessing *HTR10p::HTR10-Clover* via the *Agrobacterium tumefaciens* strain GV3101 (pMP90) by the floral dip method. According to the pKAMA-ITACHI vector system (Tsutsui and Higashiyama, 2017), T1 seeds were selected by red fluorescence and sown on MS medium. Four independent T1 lines were confirmed to exhibit the *tes* mutant phenotype, e.g., larger pollen grains and more than one pair of sperm cells in the pollen (Spielman et al., 1997; Scott et al., 2008). Putative mutations (insertions or deletions) at the targeted site of the *TES* gene were detected by genomic polymerase chain reaction and heteroduplex mobility assays. Briefly, DNA fragments were amplified with a primer pair (5′-CTCAAGAAAGGAGCTTTGTC-3′ and 5′-TGTAAATGTTGCCTGCTTTC-3′) for each T1 line, and were heat-denatured and re-annealed to form heteroduplex DNAs if mutations were induced into the sgRNA target site. We tested for a mobility shift of the heteroduplex DNAs by polyacrylamide gel electrophoresis using a 12% polyacrylamide gel. T1 lines possessing a putative mutation according to this test were selected to confirm the mutation in the T2 lines by sequence analysis. One of the obtained *tes* homozygous mutants, hereafter referred to as *tes*^*cri*^, was selected and backcrossed with the *HTR10p::HTR10-Clover* line to maintain a heterozygous mutant and keep the diploid status. Confirmation of the *tes* phenotype of the *tes*^*cri*^ mutants was performed using homozygous mutants in the selfed offspring from the heterozygous mutant.

For the phenotypic analysis of the *tes* pollen, pollen grains from *tes*^*cri*^ (*HTR10p::HTR10-Clover*) and *tes-4* (*HTR10p::HTR10-mRFP*) mutant alleles were fixed with 4% paraformaldehyde in phosphate-buffered saline (PBS). After removal of the paraformaldehyde solution, the pollen grains were dipped into 1 × PBS and observed using an upright microscope (Axio Imager.A2; Zeiss) to count the number of sperm cell pairs labeled with Clover and mRFP, respectively. Five (*tes-4*) and six (*tes*^*cri*^) plants are used to examine proportion of the pollen grains with each number of sperm cell pairs.

## Results

### Laser Irradiation of the Egg Cell Nucleus Caused Specific Damage to the Egg Cell

To study the communication between male and female gametes upon double fertilization, we attempted to disrupt each of the female gametes and performed live cell imaging of double fertilization in *A. thaliana* using a semi-*in vivo* fertilization assay (Figure 1A; Hamamura et al., 2011; Nagahara et al., 2015). Through the specific disruption of the egg cell, we examined the fertilization pattern of the central cell when the function of the other fertilization target, namely the egg cell, was eliminated. We used a femtosecond pulse laser to damage the egg cell of the ovule without damaging the surrounding cells, as this enabled disruption of a single specific cell inside the ovule (Hamamura et al., 2014; Gooh et al., 2015). Using ovules that were tagged with H2B-GFP (nuclei) and tdTomato-LTI6b (plasma membrane) fluorescent markers, we conducted laser irradiation of the egg cell nuclei and monitored the ovules for 130 s to ascertain whether the targeted egg cells were specifically disrupted (Figure 1B, Supplementary Videos 1, 2). Differential interference contrast (DIC) microscopy showed that the disrupted egg cells underwent rapid shrinking and altered shape exclusively in the ovule (Supplementary Figure 1, Supplementary Video 1). The GFP signal in the egg cell nucleus was reduced immediately after laser irradiation (Figure 1B, Supplementary Figure 1, top and middle), and the egg cell plasma membrane was deformed within 130 s, as observed by DIC microscopy (Supplementary Figure 1, bottom). Meanwhile, the remaining nuclei and the shapes of the central cell and synergid cells appeared to be intact, according to the GFP and tdTomato signals seen following this procedure (Figure 1B, Supplementary Video 2). Although deformation of the egg cell shape did not always coincide with laser irradiation, our observations indicated that irradiation by femtosecond pulse laser to the nucleus induced disruption or damage to specific cells inside of the ovule.

### The Central Cell in the Ovule With a Laser-Disrupted Egg Cell Predominantly Showed Normal Fertilization With a Single Sperm Cell

To investigate the fertilization pattern in the laser-irradiated ovules, we observed and evaluated the movement of the sperm nuclei labeled with HTR10-mRFP based on previously described methods (Hamamura et al., 2011; Nagahara et al., 2015). Following sperm cell discharge from the pollen tube to the region between the egg and central cells, sperm cell nuclei start to move toward the respective target female gamete nuclei, which is indicative of gamete membrane fusion. If the discharged sperm cells in the region between the female gametes were observed as immobile, they were considered to be incapable of fusion.

**Figure 2:**
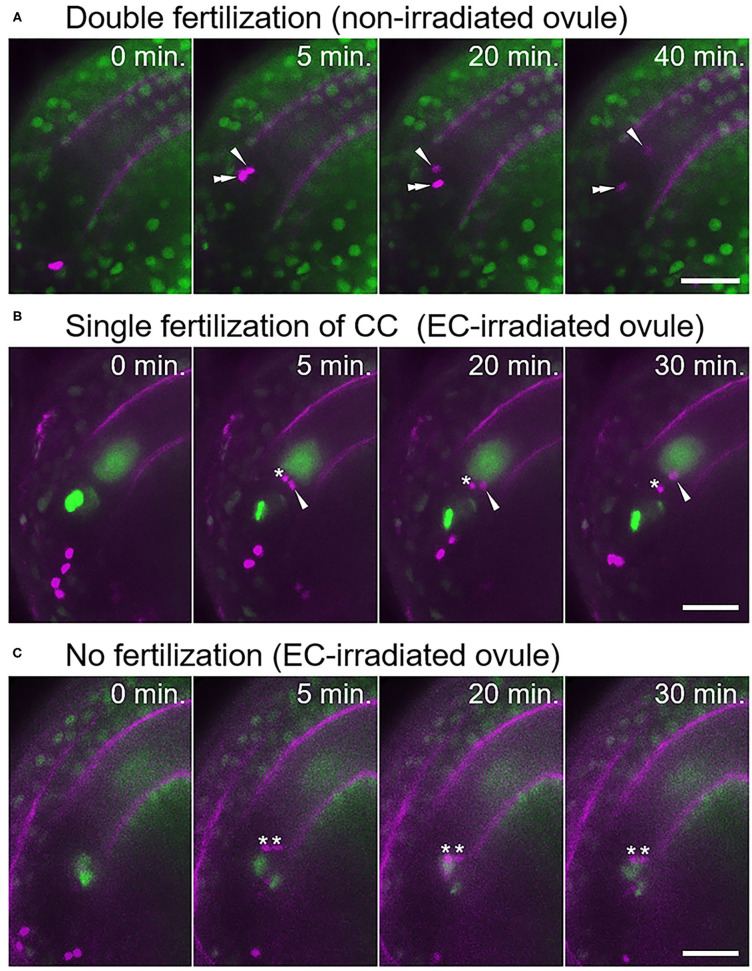
Fertilization pattern of two sperm cells released into the ovule with a laser-irradiated egg cell. **(A)** Time-series images of the fertilization process (normal double fertilization) in a non-irradiated ovule. **(B,C)** Time-series images of the fertilization process in the egg cell-irradiated ovules. **(B)** One sperm cell nucleus moved toward the central cell nucleus, and another sperm cell nucleus remained at the release position. **(C)** Two sperm cell nuclei remained at the release position, indicating unfertilized sperm cells. Zero minutes is the last frame just before pollen tube discharge. Arrowheads and double arrowheads indicate nuclei of sperm cells fertilized the central cell and the egg cell, respectively. Asterisks indicate nuclei of unfertilized sperm cells. Scale bars, 20 μm. See also Supplementary Videos 3–6, for including other examples.

Under our test conditions, double fertilization was predominantly observed in non-irradiated ovules (77%, *n* = 17; Figure 2A, Supplementary Video 3, Table 1), with few exceptions. These infrequent exceptions included single fertilization of either the egg cell (6%) or the central cell (6%), and no fertilization of both female gametes (12%), probably due to unintended damage during dissection (Table 1). In contrast, sperm cell nuclei movement followed by double fertilization was observed in only 25% of the egg cell-irradiated ovules (*n* = 40; Table 1), which was significantly lower than the frequency in non-irradiated ovules (Fisher's exact test, *p*-value = 0.0008). Among the 40 ovules, DIC observation revealed 12 ovules with deformed egg cells after laser irradiation, and only one out of these 12 ovules showed a double fertilization pattern (Table 1). Even though we did not observe obvious deformation of the egg cell in the remaining 28 ovules within 130 s, the sperm cells failed to fertilize the egg cells in 19 ovules. These results demonstrated that laser irradiation of the egg cell nucleus could cause considerable and specific damage to the egg cell, although a reduced GFP signal of the nucleus following laser irradiation could not always be linked to cell disruption.

**Table 1:** Fertilization pattern of sperm cells in laser-irradiated ovules.

**Irradiated nucleus**	**Total number of ovules**	**Frequency of fertilization pattern (%)**				
		**Double fertilization**	**Single fertilization of the central cell**	**Single fertilization of the egg cell**	**Polyspermy of the central cell**	**No fertilization**
None	17	13 (77%)	1 (6%)	1 (6%)	0 (0%)	2 (12%)
ECN	40	10 (25%)	22 (55%)	0 (0%)	1 (3%)	7 (18%)
ECN^*^ (deformed EC)	12	1 (8%)	7 (58%)	0 (0%)	1 (8%)	3 (25%)
1 SYN	12	10 (83%)	2 (17%)	0 (0%)	0 (0%)	0 (0%)
CCN	18	14 (78%)	2 (11%)	0 (0%)	2 (11%)	0 (0%)

*ECN, egg cell nucleus; SYN, synergid cell nucleus; CCN, central cell nucleus*.

* *ECN (deformed EC) represents a part of ECN, of which irradiated egg cell (EC) was deformed in 130 s as mentioned in Results*.

In the egg cell-irradiated ovules, single fertilization of the central cell was predominantly observed (55%; Figure 2B, Supplementary Video 4, Table 1), which was significantly higher than the frequency in non-irradiated ovules (Fisher's exact test, *p*-value = 0.0004). This type of single fertilization was observed in seven (58%) of the 12 egg-disrupted ovules with obvious egg deformation. Although one remaining sperm cell was observed at the release position, which appeared to contact both of the female gametes (Figure 2B). However, polyspermy of the central cell occurred only exceptionally (3%; Supplementary Figure 2, Supplementary Video 5, Table 1). A few ovules failed to fertilize with both sperm cells (18%; Figure 2C, Table 1), a similar proportion to the control ovules under our test conditions (Table 1). Our results indicate that the central cell preferentially executes membrane fusion with only one of the two sperm cells, even when the other sperm cell is positioned in close proximity, as it was unable to fertilize the egg cell.

### Laser Irradiation of the Central Cell Nucleus Affected Karyogamy but Not Plasmogamy With the Sperm Cell

We also evaluated the effect of irradiation of the nuclei of the central cell and the synergid cell with a femtosecond pulse laser. In ten out of 12 synergid cell-irradiated ovules, though the frequency of disruption was unclear, normal double fertilization was predominantly observed at the same frequency as in the control ovules (83%; Table 1). This suggested that laser irradiation did not cause much damage to the neighboring egg cell and that damage to one of two synergid cells did not affect the fertilization processes. When laser irradiation of the central cell caused a change in membrane shape, severe damage to the entire female gametophyte was always observed. Thus, we assessed the fertilization pattern in the ovules with a reduced GFP signal of the central cell nucleus but not where the plasma membrane shape was altered (Figure 1C). In most of the central cell-irradiated ovules (78%), two sperm cell nuclei moved toward the egg and the central cell nucleus, respectively, indicating that gamete membrane fusion occurred in both of the female gametes at near-normal frequencies (Supplementary Figure 3, Table 1). However, consistent with laser irradiation of the nucleus, sperm cell nuclei that entered into a laser-irradiated central cell sometimes roamed around the nuclear region (*n* = 11 out of 18 irradiated ovules; Supplementary Video 6). This result suggested that laser irradiation of the central cell nucleus was sufficient to inhibit nuclear fusion. Single fertilization (11%) and no fertilization (0%) were observed less frequently (Table 1) and were comparable with those of the control ovules. Therefore, we suggest that the damage to each nucleus and cell was specific and targeted.

### Excessive Sperm Cells Did Not Always Lead to Polyspermy of the Central Cell

Next, we investigated what would happen if a single pollen tube delivered more than two sperm cells. We examined the behavior when more than two sperm cells were released to the region between the egg and central cells at one time. The pollen grains of *tes* mutants contain variable numbers of sperm cells, ranging from two to eight (Spielman et al., 1997; Scott et al., 2008). In *tes* mutant pollen tubes, multiple sperm cells are formed as multiple sets of two sperm cells (Scott et al., 2008). For example, four sperm cells are likely to be formed from two sets of sperm cell pairs. We generated knockout mutants for the *TES* gene in a HTR10-Clover line by CRISPR/Cas9-mediated genome editing (referred to as *tes*^*cri*^), and in a HTR10-mRFP line by crossing with a *tes-4* mutant (Spielman et al., 1997) (Supplementary Figure 4A). First, we checked the number of sperm cell pairs in the pollen of each mutant and found pollen grains with one or two sperm cell pairs (two or four sperm cells) in both mutants (Supplementary Figures 4B,C). We rarely observed pollen grains with three sperm cell pairs (six sperm cells), and we found no pollen grains with four pairs (eight sperm cells) under our test conditions (Supplementary Figure 4C).

We performed a semi-*in vivo* fertilization assay using both of the *tes* homozygous mutant lines as male donors. We observed 59 ovules in total that accepted sperm cells from the *tes* pollen tube (*tes*^*cri*^, *n* = 39; *tes-4, n* = 20). Based on the fact that the majority of pollen possessed one sperm cell pair (Supplementary Figure 4C), we often observed ovules that accepted *tes* pollen tubes with two sperm cells (41/59; Supplementary Table 1) and normal double fertilization in the egg and central cells (35/41; Supplementary Figure 5). Therefore, we considered that the fertilization ability of the *tes* sperm cells was normal.

**Figure 3:**
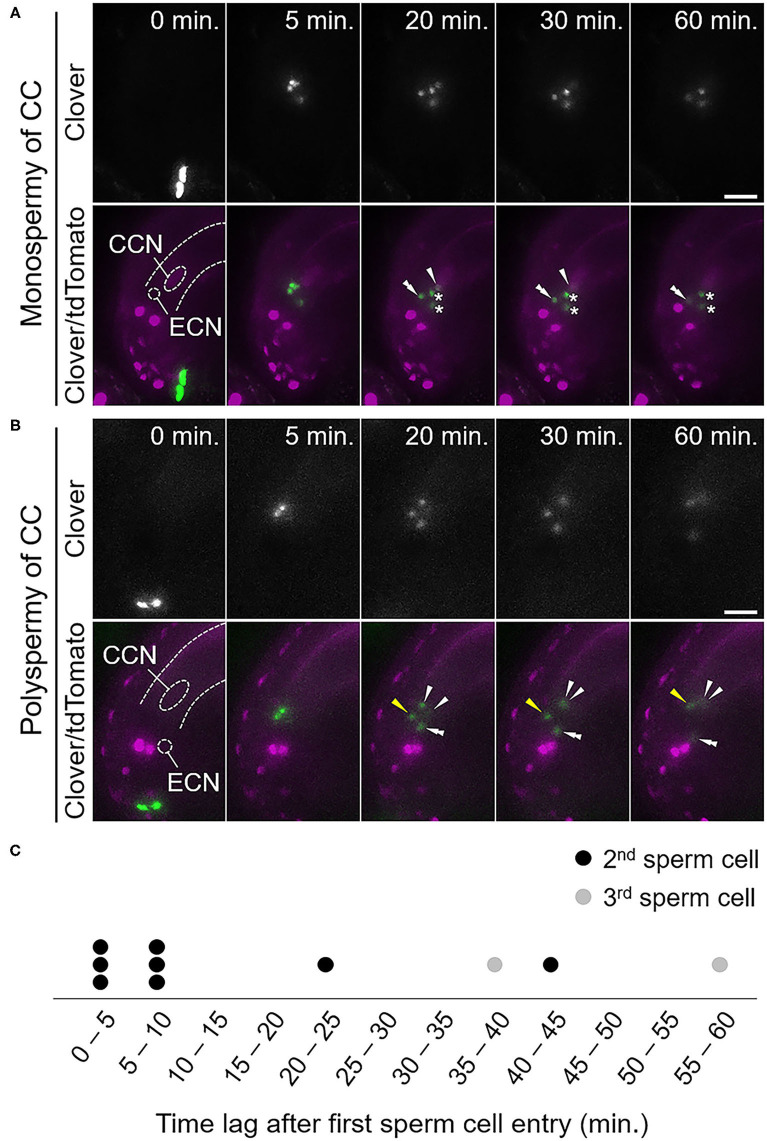
Fertilization pattern of excessive sperm cells released from a pollen tube of a *tes* mutant. **(A,B)** Time-series images of the fertilization process of ovules receiving four sperm cells from a pollen tube of the *tes*^*cri*^ mutant. **(A)** Among the four sperm cell nuclei, one moved toward the egg cell nucleus and another one moved toward the central cell nucleus. The other two sperm cell nuclei remained at the release position, indicating unfertilized sperm cells. **(B)** Among the four sperm cell nuclei, one moved toward the egg cell nucleus and the other three moved toward the central cell nucleus, indicating polyspermy of the central cell. The third sperm cell nucleus (yellow arrowhead) moved toward the central cell nucleus behind the other two sperm cell nuclei. Zero minutes is the last frame just before pollen tube discharge. CCN, central cell nucleus; ECN, egg cell nucleus; dashed lines, outline of the embryo sac. **(C)** Time plot of sperm cell movement after the first sperm cell movement toward the central cell nucleus. Circles indicate individual sperm cells that moved second (black) and third (gray). Scale bars, 20 μm. Arrowheads and double arrowheads indicate nuclei of sperm cells fertilized the central cell and the egg cell, respectively. Asterisks indicate nuclei of unfertilized sperm cells. See also Supplementary Figures 5–7 and Supplementary Videos 7, 8.

Among 14 ovules receiving four sperm cells from the *tes* pollen tube (*tes*^*cri*^, *n* = 7; *tes-4, n* = 7; Supplementary Table 1), we identified two patterns of central cell fertilization: monospermy and polyspermy (Table 2, Figure 3). In seven out of 14 ovules (50%), each of two sperm cells fused with the egg and central cells, respectively, whereas the remaining two sperm cells remained at the release position (monospermy of the central cell; Figure 3A, Supplementary Figure 6A, Table 2, Supplementary Video 7). In six out of 14 ovules (43%), more than one sperm cell fertilized the central cell (polyspermy of the central cell; Figure 3B, Supplementary Figure 6B, Table 2, Supplementary Video 8). We observed other four cases in which ovules received a pollen tube with six sperm cells, and all four showed polyspermy in the central cell (100%, Supplementary Figure 7, Table 2). The more sperm cells are delivered simultaneously, the more the possibility of polyspermy increased, although the difference was not statistically significant possibly due to low sample size. Among total ten cases of polyspermy of the central cell by *tes* pollen tubes (with 4 and 6 sperm cells delivered), we observed two ovules of which central cell accepted three sperm cells (Figures 3B,C, Supplementary Video 8). In contrast, we observed no polyspermy of the egg cell. Thus, live imaging confirmed the strict polyspermy block of the egg cell at the step of gamete fusion, consistent with previous findings of ploidy analysis of embryos in *tes* mutants (Scott et al., 2008). Furthermore, we showed that the central cell displayed a moderate polyspermy block mechanism when the ovule received excessive sperm cells.

**Table 2:** Fertilization pattern of excessive (four or six) sperm cells released into the ovules.

**Mutant allele**	**Total number of ovules**	**Number of fertilization pattern**		
		**Monospermy of the central cell**	**Polyspermy of the central cell**	**Single fertilization of the egg cell**
*tes^*cri*^*	10 (3)	5	5 (3)	0
*tes-4*	8 (1)	2	5 (1)	1
total	18 (4)	7	10 (4)	1

*The number of ovules receiving six sperm cells is shown in parentheses*.

To examine the temporal dynamics of the polyspermy events in the central cell, we monitored the time between the first and the following sperm nucleus movements toward the central cell nucleus. Among ten sperm cells from eight polyspermy events in the central cell that were entirely captured from the sperm cell discharge, four of the additional sperm cell nuclei entered the central cell 20–60 min after fertilization by the first sperm cell (Figures 3B,C, Supplementary Video 8). The remaining six sperm cell nuclei entered almost simultaneously, or within 10 min after the first sperm cell fertilization (Figure 3C). Together with our observation of partial polyspermy of the central cell, we saw that polyspermy block in the central cell was incomplete *in vivo* during 60 min after fertilization of the first sperm cell.

## Discussion

Angiosperms are unique to produce the two adjacent dimorphic female gametes, the egg and central cells (Li and Yang, 2020). Polyspermy in the egg cell risks the production of a sterile triploid plant in return for the chance of polyploidization and speciation (Toda et al., 2016; Nakel et al., 2017; Mao et al., 2020). Although the central cell gives rise to the nutritious endosperm after fertilization and does not contribute to the genome of the next generation, polyspermy in the central cell results in a paternal and maternal genome imbalance in the endosperm and disturbs normal seed development (Scott et al., 2008; Köhler et al., 2010). Thus, precise regulation for one-to-one fertilization in both of the female gametes is important for successful double fertilization and proper seed development in angiosperms. Previous studies showed the presence of cellular mechanisms of the egg cell to prevent additional sperm cell entry and the dynamic movement of two sperm cells released at the region between the egg and central cells (Kranz et al., 1995; Hamamura et al., 2011). Nevertheless, it was unclear how angiosperm double fertilization was accomplished by four gametes in the ovule without polyspermy of both female gametes. In this study, we investigated the fertilization dynamics when supernumerary sperm cells were released into an ovule using *A. thaliana* and a semi-*in vivo* live imaging system. We showed that not only the egg cell but also the central cell possess mechanisms to block secondary sperm cell entry (Figures 2B, 3A). Since this mechanism worked in cases when the central cell received two sperm cells without an intact neighboring egg cell, and where there were excessive sperm cells in an intact ovule, the central cell in the ovule might possess a cellular mechanism to reduce the risk of polyspermy, independent of the egg cell. The fact that the polyspermy block mechanism of the central cell was incomplete supports a previous report that polyspermy block in the central cell is less strict than in the egg cell (Scott et al., 2008). Our results provide additional information on the actual frequency and timing of the polyspermy events, which will be useful for further analyses to understand the spatiotemporal control of one-to-one fertilization in the ovule.

Femtosecond pulse laser technology has been used in several studies to dissect a single organelle or neuron in living animal cells or tissues and to analyze early embryo development of *A. thaliana* by inactivation of single specific cell types (Watanabe et al., 2009; Gooh et al., 2015). This study demonstrated the use of femtosecond pulse laser technology to inactivate specific cells inside plant tissues, which enabled us to examine the fertilization processes of female gametes (Figure 1). We successfully inhibited the fertilization of the egg cell by laser irradiation of the egg cell nucleus, as the egg cells failed to fertilize in 75% of the egg cell-irradiated ovules (Table 1). Disruption or damage of the egg cell did not affect central cell fertilization. Although 18% of the egg cell-irradiated ovules showed fertilization defects of both sperm cells, characteristic of *gcs1/hap2* sperm cells (Figure 2C; Mori et al., 2006; von Besser et al., 2006; Hamamura et al., 2011), this occurred in a similar proportion to the control ovules. The egg-secreted EC1 peptides play essential roles in double fertilization by activating sperm cells to redistribute the fusogen GCS1/HAP2 to the cell surface (Sprunck et al., 2012). The EC1 peptides are suggested to be released from the egg cell upon the arrival of the sperm cell and are thought to be important for sperm cell adhesion and separation (Sprunck et al., 2012; Cyprys et al., 2019). Our results showed that sperm cell fusion in the central cell was not much affected in most of the egg cell-irradiated ovules (Figure 2B, Table 1). A small amount of the EC1 peptide, which accumulated either before the arrival of the sperm cell or was released from the laser-irradiated egg cell due to residual activity, might suffice for fertilization of the central cell. To clarify the contribution of the EC1 peptides to central cell fertilization, the dynamics of EC1 secretion, as well as the fertilization process, should be addressed by live cell imaging.

We were able to disrupt the egg cells, as we observed shrinking of the egg cell and some unfertilized sperm cells in the egg cell-irradiated ovule (Supplementary Figure 1, Figure 2). We were also able to damage the central cell, although this suffered less damage than the egg cell under our test conditions. This was because we were unable to severely damage the central cell without causing the collapse of the entire female gametophyte. Similar observations for the central cell have also been reported in laser ablation of protruding female gametophytes of *Torenia fournieri* (Higashiyama et al., 2001). It should be noted that the plasma membrane of some egg cells remained functional since normal double fertilization behavior was still observed. It is proposed that sperm membrane-localized DMP8 and DMP9 are important for sperm–egg fusion (Takahashi et al., 2018; Cyprys et al., 2019). It would be interesting to note whether central cell fertilization in egg cell-irradiated ovules is altered when receiving sperm cells of mutants for DMP8 and DMP9. Nevertheless, our observation implies that polyspermy block would function in the central cell independent of egg cell fertilization. In other words, the central cell might have a cell-autonomous mechanism to accept only one of two sperm cells delivered by a pollen tube.

The molecular basis of polyspermy block remains to be defined in plants. Calcium spikes occur immediately after gamete fusion, and these primarily occurred in the egg cell and in half of the central cells (Denninger et al., 2014; Hamamura et al., 2014). Incomplete generation of a calcium spike in the central cell might correlate with central cell polyspermy, which was observed in approximately half of the ovules receiving excessive *tes* mutant sperm cells (Table 2). An influx of calcium ions is thought to induce changes in the properties of the zygote, such as cell wall formation (Kranz et al., 1995; Antoine et al., 2001). Although it remains unclear whether rapid cell wall formation also occurs in the central cell immediately after fertilization, it is important to establish the relationship between the calcium dynamics and polyspermy of the central cell. The central cell has a very thin cell wall and is thus possibly ready to fuse with the persistent synergid cell immediately after fertilization, to avoid polytubey (Maruyama et al., 2015). The supply of more than two sperm cells by a single pollen tube, such as the *tes* mutant pollen, would be rare, whereas polytubey occurs with relatively high frequency (>2%) (Beale et al., 2012; Kasahara et al., 2012; Maruyama et al., 2013; Völz et al., 2013). Therefore, the central cell could play a role in polytubey block through synergid–endosperm fusion rather than polyspermy block with the thin cell wall. After the cell fusion of the first sperm, the surface of the fertilized central cell might be permissive, unlike the fertilized egg cell, and able to fuse with an additional sperm cell, at least within 1 h (Figure 3C), and with the persistent synergid cell within several hours (Maruyama et al., 2015).

We noticed that a secondary sperm cell entered the central cell almost simultaneously, or soon after the first sperm cell entry, in approximately half of the polyspermy events (Figure 3C). This shows that accidental polyspermy can frequently occur upon membrane fusion of the central cell when excessive sperm cells are delivered into the ovule. Surprisingly, in the ovules that accepted six sperm cells, one sperm cell fertilized the egg cell, two sperm cells fertilized the central cell, and the remaining three sperm cells were not accepted by either of female gametes (Supplementary Figure 7). These observations imply that temporal and spatial restriction, i.e., the acceptance of the sperm cells for a limited time and position, is important for successful double fertilization. This is consistent with our observation that polyspermy was more frequently observed in ovules accepting excessive sperm cells from *tes* pollen tubes than egg-irradiated ovules accepting two sperm cells from a wild-type pollen tube (Tables 1, 2). The frequency of polyspermy increased as the number of delivered sperm cells increased. Further analysis of the release position of the supernumerary sperm cells, as well as of cellular responses such as the calcium dynamics, will further aid our understanding of the one-to-one fertilization mechanism through the restriction of central cell polyspermy, which is mediated by limiting the location and timing of fertilization.

## Data Availability Statement

The raw data supporting the conclusions of this article will be made available by the authors, without undue reservation.

## Author Contributions

SN and TH designed this work. SN conducted all experiments in this work with advice from HT. SN wrote the manuscript. HT and TH conducted proofreading of the manuscript. All authors contributed to the article and approved the submitted version.

## Conflict of Interest

The authors declare that the research was conducted in the absence of any commercial or financial relationships that could be construed as a potential conflict of interest. The handling editor declared a past co-authorship with the authors SN and TH.
