# Supplementary material for: Polyspermy Block in the Central Cell During Double Fertilization of Arabidopsis thaliana
Source: Front Plant Sci. 2021 Jan 12;11:588700. doi: 10.3389/fpls.2020.588700 (PMC7835324; doi:10.3389/fpls.2020.588700)
Supplement: Supplementary file 1 [file Data_Sheet_1.docx]

Supplementary Material

# Supplementary Figures


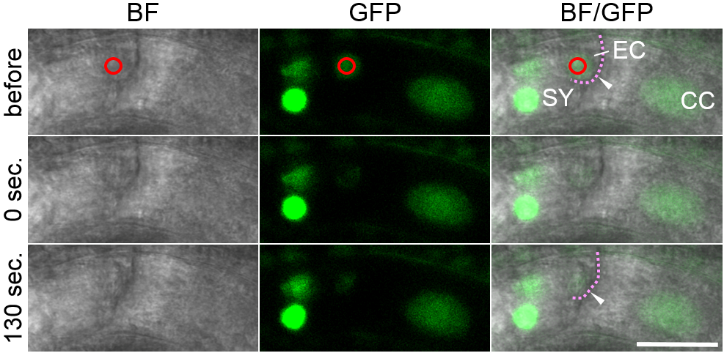


**Supplementary Figure S1.** Laser irradiation to an egg cell nucleus within the ovule. Bright-field (BF, left), fluorescent (GFP, middle), or merged (right) images of a part of the embryo sac before and after laser irradiation to the egg cell. Nuclei of female gametophytic cells were labeled with GFP. Red circles indicate the laser-irradiated regions, consistent with the egg cell nucleus. Images are before, just after (0 s) and 130 s after irradiation. Arrowheads indicate intact (before) and deformed (130 sec) egg cell membranes (pink dashed lines). Scale bar, 20 µm. EC, egg cell; CC, central cell; SY, synergid cell. See also Supplementary Video S1.


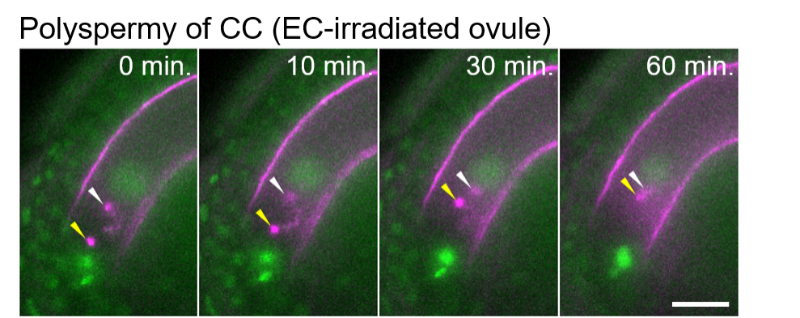


**Supplementary Figure S2.** Live cell imaging of the fertilization process in the ovule with the irradiated egg cell nucleus. Two sperm cell nuclei (arrowheads) moved toward the central cell nucleus, showing polyspermy of the central cell. 0 minutes indicates onset of time-lapse imaging. Scale bar, 20 µm. See also Supplementary Video S5.


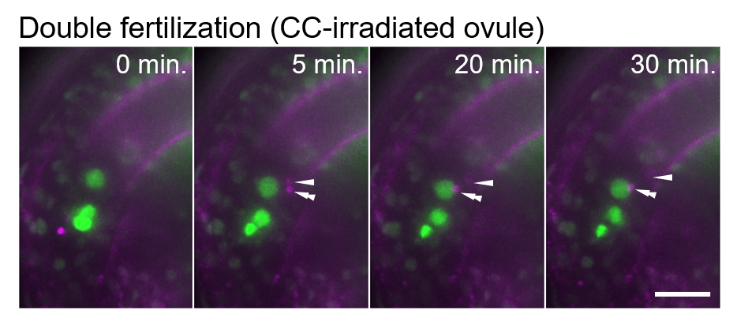


**Supplementary Figure S3.** Live cell imaging of the fertilization process in the ovule with the irradiated central cell nucleus. One sperm cell nucleus moved toward the central cell nucleus (arrowhead), and another sperm cell nucleus moved toward the egg cell nucleus (double arrowheads). 0 minutes is the last frame just before pollen tube discharge. Scale bar, 20 µm. For another example, see also Supplementary Video S6.


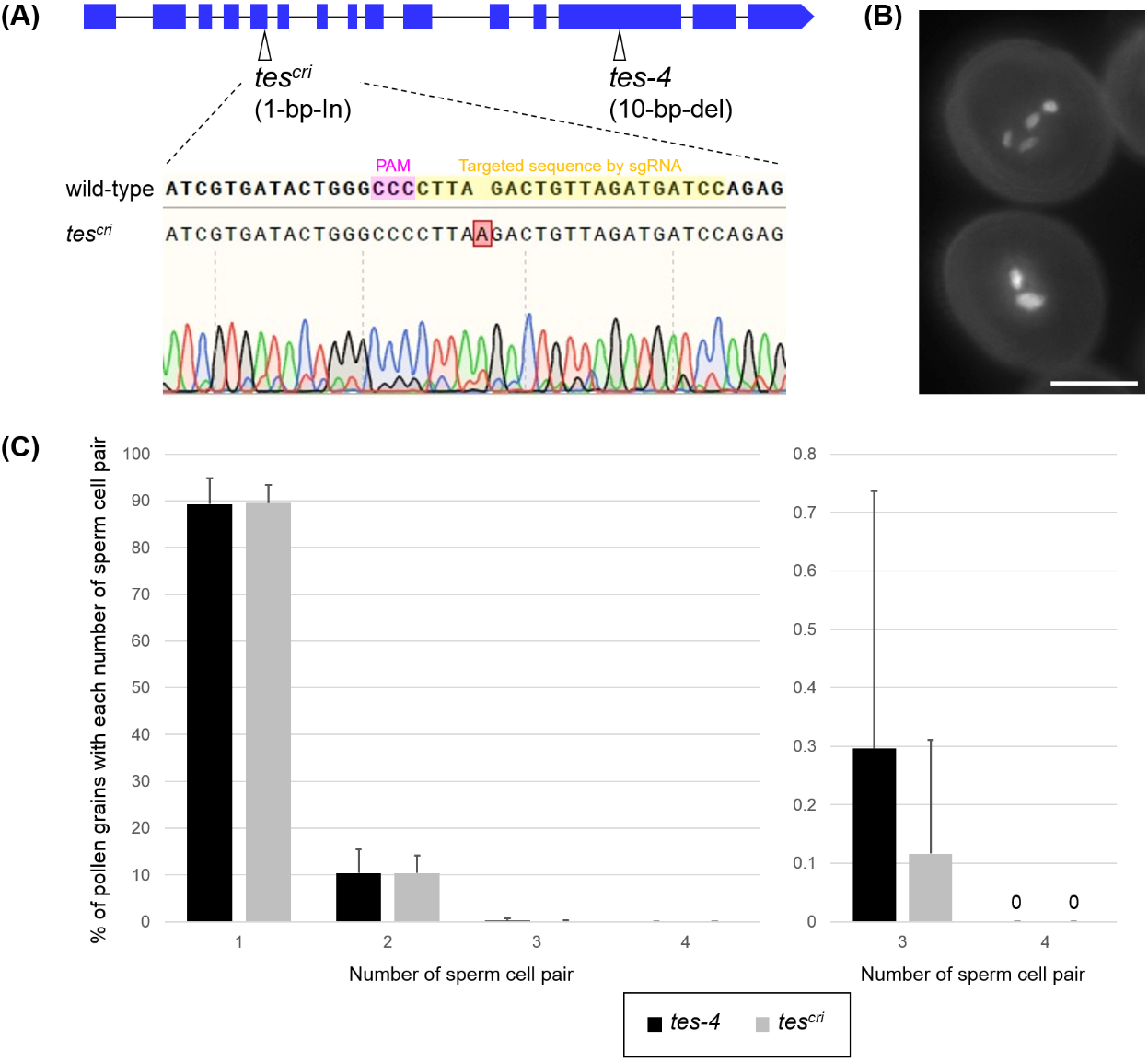


**Supplementary Figure S4.** Comparison of the two *tetraspore* mutants. **(A)** Schematic representation of the region of mutation in the *tes^cri^* and *tes-4* mutants on the *TETRASPORE* gene. The bottom image represents part of the sequencing result of the *tes^cri^* mutant. The target sequence by single guided RNA (sgRNA) and a protospacer adjacent motif (PAM) for CRISPR/Cas9 system are highlighted in the wild-type sequence with yellow and pink, respectively. **(B)** Pollen grains with two (four sperm cells, top) or one (two sperm cells, bottom) pair of sperm cells of the *tes^cri^* mutant. Sperm cell nuclei were visualized by HTR10-Clover. Scale bar, 20 µm. **(C)** Proportion of the pollen grains with each number of sperm cell pairs in the *tes-4* (black) and *tes^cri^* (gray) mutants. The right graph shows the magnification of the results of pollen grains with three or four sperm cell pairs. Note that we observed no pollen grains with more than three pairs (six sperm cells) under our test conditions. Data are the average and standard deviations from five (*tes-4*) and six (*tes^cri^*) plants. In each plant, 405–602 pollen grains were examined.


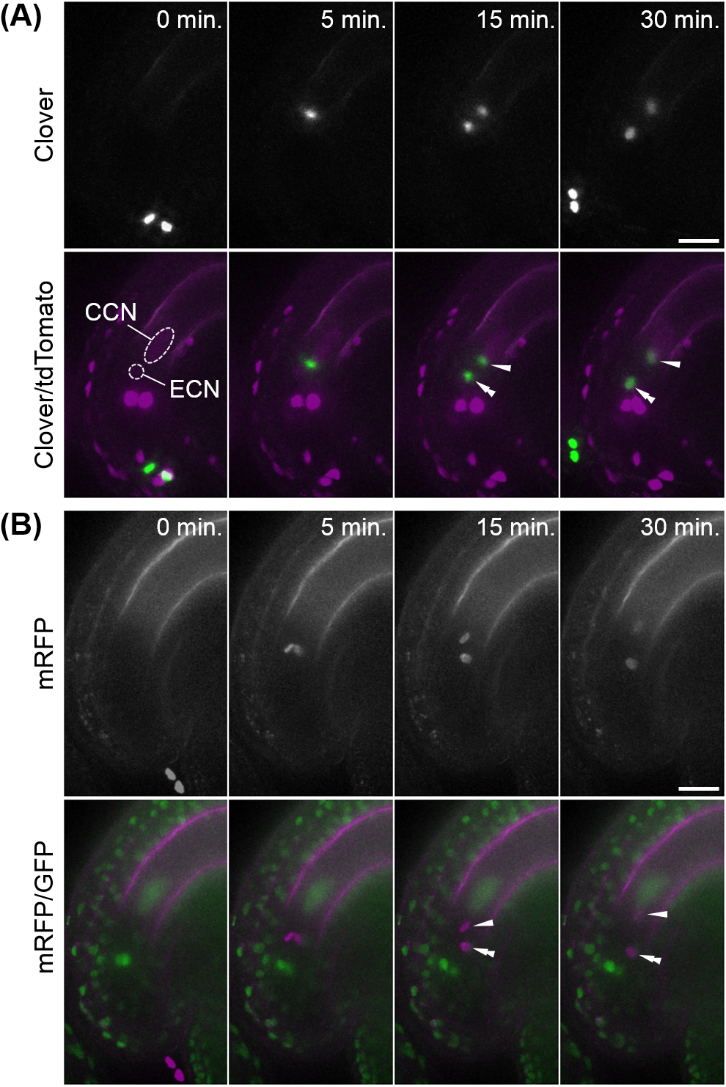


**Supplementary Figure S5.** Live cell imaging of the fertilization process of two sperm cells from the *tes* pollen. **(A, B)** Time-series images of the fertilization process of two sperm cells from the *tes^cri^* **(A)** and *tes-4* **(B)** pollen. One sperm cell nucleus moved toward the central cell nucleus (arrowhead), and another sperm cell nucleus moved toward the egg cell nucleus (double arrowheads). 0 minutes is the last frame just before pollen tube discharge. Scale bars, 20 µm. CCN, central cell nucleus; ECN, egg cell nucleus.


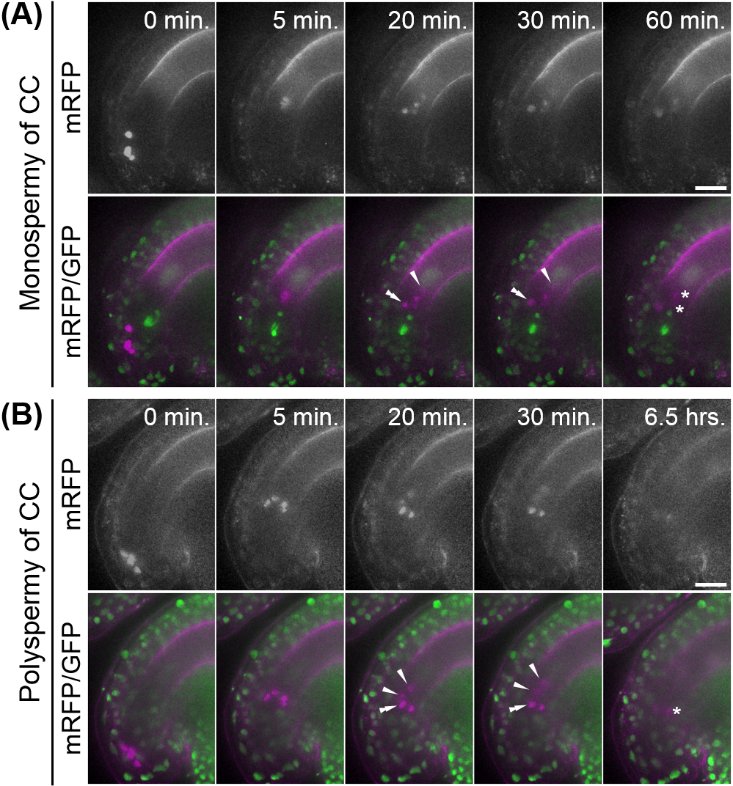


**Supplementary Figure S6.** Fertilization pattern of the excessive sperm cells from *tes-4* pollen. **(A, B)** Time-series images of the fertilization process of four sperm cells from the *tes-4* pollen. **(A)** Among the four sperm cell nuclei, one moved toward the egg cell nucleus (double arrowheads) and another one moved toward the central cell nucleus (arrowhead). The other two sperm cell nuclei remained at the release position, indicating unfertilized sperm cells (asterisks). **(B)** Among the four sperm cell nuclei, one moved toward the egg cell nucleus (double arrowheads), and two moved toward the central cell nucleus (arrowheads), indicating polyspermy of the central cell. The other one remained at the release position, indicating an unfertilized sperm cell (asterisk). 0 minutes is the last frame just before pollen tube discharge. Note that the last frame in **(B)** was acquired at 6 h after the fourth frame, showing an unfertilized sperm cell (asterisk). Scale bars, 20 µm. See also Figures 3A, 3B for examples of the *tes^cri^* mutant.


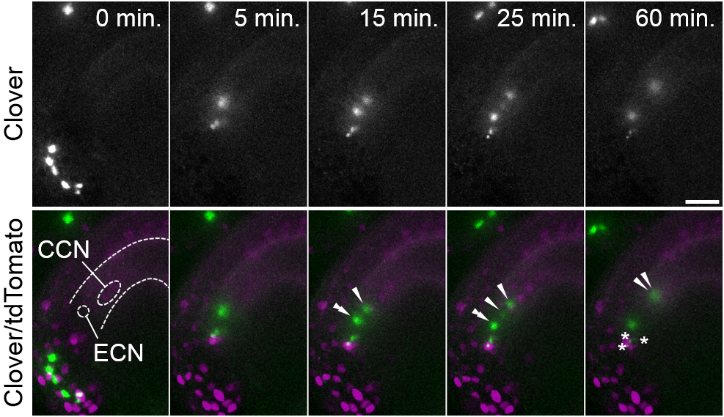


**Supplementary Figure S7.** Fertilization pattern of six sperm cells from *tes^cri^* pollen. Time-series images of the fertilization process of six sperm cells from the *tes^cri^* pollen. Among the six sperm cell nuclei, one moved toward the egg cell nucleus (double arrowheads), and two moved toward the central cell nucleus (arrowheads), indicating polyspermy of the central cell. The remaining three sperm cell nuclei stayed at the release position (asterisks). 0 minutes is the last frame just before pollen tube discharge. Scale bars, 20 µm. CCN, central cell nucleus; ECN, egg cell nucleus; dashed lines, outline of the embryo sac.

**Supplementary Table S1.** The number of ovules receiving *tetraspore (tes)* sperm cells.


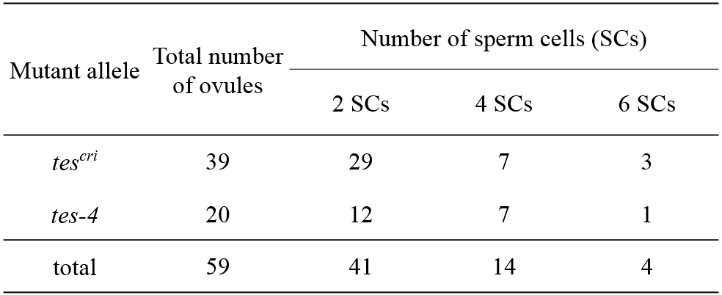


# Supplementary Videos

**Supplementary Video S1.** Time-series images corresponding to Supplementary Figure S1. A white arrowhead indicates the deformed egg cell membrane. Scale bar, 20 µm.

**Supplementary Video S2.** Time-series images corresponding to Figure 1B. A white arrowhead indicates the deformed egg cell membrane. Scale bar, 20 µm.

**Supplementary Video S3.** Time-series images corresponding to Figure 2A. Two sperm cell nuclei (two magenta-fluorescent foci) moving toward the respective target nuclei are shown as a typical case of normal double fertilization in a non-irradiated ovule. Scale bar, 20 µm.

**Supplementary Video S4.** Time-series images corresponding to Figure 2B. A yellow arrowhead indicates sperm cell nuclei moving toward the central cell nucleus. A white arrowhead indicates the sperm cell nucleus remaining at the release position. Scale bar, 20 µm.

**Supplementary Video S5.** Time-series images corresponding to Supplementary Figure S2. Note that two sperm cell nuclei (two magena-fluorescent foci) are in the central cell, showing polyspermy as an exceptional case in an egg-irradiated ovule. 0 minutes indicates the onset of time-lapse imaging. Scale bar, 20 µm.

**Supplementary Video S6.** Time-series images of sperm cell nuclei just after fertilization in the central cell-irradiated ovule. White and yellow arrowheads indicate sperm cell nuclei moving toward the central cell nucleus and the egg cell nucleus, respectively. Note that the sperm cell nucleus entered into the central cell nucleus and roamed around the nucleus region, as confirmed by autofluorescence. Time indicates elapsed time. Scale bar, 20 µm.

**Supplementary Video S7.** Time-series images corresponding to Figure 3A. White and yellow arrowheads indicate the sperm cell nuclei moving toward the central cell nucleus and the egg cell nucleus, respectively. Scale bar, 20 µm.

**Supplementary Video S8.** Time-series images corresponding to Figure 3B. White and yellow arrowheads indicate the sperm cell nuclei moving toward the central cell nucleus and the egg cell nucleus, respectively. Scale bar, 20 µm.
